# “The Smartphone’s Guide to the Galaxy”: In Situ Analysis in Space

**DOI:** 10.3390/bios8040096

**Published:** 2018-10-19

**Authors:** Joost Nelis, Christopher Elliott, Katrina Campbell

**Affiliations:** Institute for Global Food Security, School of Biological Sciences, Queen’s University Belfast, David Keir Building, Stranmillis Rd, Belfast BT9 5AG, UK; J.Nelis@qub.ac.uk (J.N.); chris.elliott@qub.ac.uk (C.E.)

**Keywords:** smartphone, biosensor, space, analysis, health

## Abstract

A human mission to Mars can be viewed as the apex of human technological achievement. However, to make this dream a reality several obstacles need to be overcome. One is devising practical ways to safeguard the crew health during the mission through the development of easy operable and compact sensors. Lately, several smartphone-based sensing devices (SBDs) with the purpose to enable the immediate sensitive detection of chemicals, proteins or pathogens in remote settings have emerged. In this critical review, the potential to piggyback these systems for in situ analysis in space has been investigated on application of a systematic keyword search whereby the most relevant articles were examined comprehensively and existing SBDs were divided into 4 relevant groups for the monitoring of crew health during space missions. Recently developed recognition elements (REs), which could offer the enhanced ability to tolerate those harsh conditions in space, have been reviewed with recommendations offered. In addition, the potential use of cell free synthetic biology to obtain long-term shelf-stable reagents was reviewed. Finally, a synopsis of the possibilities of combining novel SBD, RE and nanomaterials to create a compact sensor-platform ensuring adequate crew health monitoring has been provided.

## 1. Introduction

### 1.1. Need for Miniaturized Sensors for Future Space Missions

Scott Kelly and Mikhail Korniyenko spent 342 days in orbit on the international space station (ISS). Their achievement shows that long-term space flights are feasible and brings humanity one step closer to one of the biggest scientific challenges of this century: Human settlement on other worlds, with the most ambitious endeavor being a human mission to Mars. Significant resources are invested in the technological advancement of rocket science in order to make this dream a reality. However, some other important facets of the challenge should not be forgotten. One of these is a better understanding of the effects of a prolonged stay in space to one’s health. For instance, it has been found that microgravity can lead to muscle atrophy after only a few weeks in space [1]. Moreover, decreased oxygen consumption during space flight can lead to a decrease in exercise capacity and might affect performance upon arrival [2]. In addition, a significant increased risk for renal stones has been reported [3] and is mainly subscribed to increased turnover of bone minerals due to bone atrophy [4]. Another possible threat to crew health is the emergence of infectious diseases. Indeed, it should not be forgotten that where humans go, microbes go. A study on the presence of microbes on a space shuttle has shown that the amount of colony forming units (CFUs) in the shuttle air increased by 300% within 12 days [5]. Moreover, it has been shown that microgravity can actually increase the growth rate and secondary metabolite production of microbes [6] and that the susceptibility of opportunistic pathogens to antibiotics as well as their virulence may change aboard the space ship [7,8]. Next to this, it has been reported that space travel can reduce the efficiency of the immune system, increase cytokine blood plasma levels, and cause reactivation of Herpes virus (HV) [9], including Latent Epstein–Barr Virus, which can lead to infectious mononucleosis [10]. Moreover, increased exposure to radiation and stress can lead to a higher risk of cancer. Indeed, all the health risks mentioned above are included in the NASA human research roadmap (https://humanresearchroadmap.nasa.gov/) and will need to be closely monitored during any long term space mission [4]. Evidently, strict monitoring of the physical and biochemical parameters as indicators of the crew health during a mission or simulated events by use of portable analytical tools could improve the understanding of these biological phenomena and enable early diagnosis, treatment and intervention measures aboard the spacecraft. Similarly, continuous environmental monitoring of both inorganic and organic compounds present in the air, water and other surfaces as well as other systems such as waste/feces and biological life support of a spacecraft can help prevent the growth of pathogenic microbes on board. Requirements for such devices, as stated by NASA in the Human Research Program Requirements Document HRP 47052 Revision E, are especially low mass, volume and power consumption. Moreover, the devices should be reliable and durable, whilst avoiding laborious analysis with bulky instruments in microgravity. Proof of concept studies for the accurate detection of microbes in space using miniature devices have already been reported [11,12]. These include the use of a miniaturized PCR system [12] and a portable DNA sequencing device based on nucleotide recognition through conformational changes in the protein-based pore (Nanopore technology) [13]. The latter was used successfully for rapid microorganism identification and possible disease diagnostics through DNA sequencing was suggested. Miniaturized Gram positive/negative bacterial and fungal detection aboard the ISS using the Lab-On-a-Chip Application Development Portable Test System (LOCAD-PTS) has also been reported as a successful system for the quantification of microorganisms aboard a spacecraft [14]. Other such miniaturized devices have been developed to perform screening tests for the detection of extra-terrestrial life. Some examples are: an automated microfluidic device using capillary electrophoresis and laser induced fluorescence for amino acid detection [15], an antibody microarray for biomolecule detection [16] and an in situ DNA sequencing device based on Nanopore technology [17]. Although these sensors do not deliver pertinent conclusions regarding the discovery of extra-terrestrial life, they could filter out which samples are potentially interesting for more detailed sophisticated analysis back on earth (https://ntrs.nasa.gov/search.jsp?R=20180002121).

### 1.2. Smartphone Based Devices for Facile Crew Health Monitoring during Deep Space Missions

The development of portable lab on a chip (LOC) or point of care (POC) bio-sensing devices is currently experiencing a major boost in many sectors, including environmental science [18], animal and human health monitoring, disease diagnosis, and food safety monitoring [19,20]. An interesting development is the use of smartphone-hyphenated biosensors that use the phone’s built in sensors and hardware to directly analyze the sample in situ. Phones nowadays are equipped with a plethora of sensors including cameras, with ever increasing resolution, and ever more sophisticated processing units, memory storage capabilities and connectivity, all in a highly compacted design. So why not utilize this? In other words, why not benefit from the already optimized miniaturization of smartphones for the further development of sophisticated sensing devices. As previously mentioned, similar systems (in terms of compactness and simplicity), are already being developed by NASA such as the mentioned LOCAD system [14,21] and water monitoring systems for in-flight microbial contamination [22]. Such systems are being developed to enable more in-flight analysis instead of relying on analysis on the ground using bulky bench-top instruments, which is often still the case [22]. However, these systems are not capable to detect between species making it impossible to distinguish pathogens from rather harmless species. Moreover, more elegant solutions using SBDs, which might be able to replace current bench-top instruments for direct inflight analysis, might already exist. In fact, one could bring this question even one step further i.e., why not piggyback already existing smartphone based sensing devices developed for biochemical sensing on Earth, for deep space missions? Indeed, such devices are often developed for use as robust and simple point of care devices in remote locations and, as such, inherently meet miniaturization and reduced power requirements. Moreover, additional costs implicated in the development of novel sensors complete with processing and memory units can be avoided by adopting currently developed systems. Thus, research in this direction seems a logical choice, if adequate robustness and sensitivity can be reached. Studies have shown that these devices can be quite sensitive. Long et al., for instance, compared the performance of a SBD spectrometer against conventional bench-top analyzers for quantifying analyte concentrations using two commercial assays based on transmission/reflection measurements (ELISA), or fluorescence intensity measurements [23]. This SBD uses either the flashlight or an integrated green laser diode for illumination of the sample which is held in a microfluidic chamber. Emerging light is then piped to the rear camera with fiber optic cable. The camera is covered by a diffraction grating which generates spectra when images are collected. The authors found that the SBD was able to predict analyte concentrations as accurately as bench-top analyzers and, in some cases, even outperform the latter. Moreover, the finding that SBDs can perform equally to bench-top instruments is not limited to a single study. Ludwig et al., tested a fluorescent protein micro array SBD for the detection of recombinant bovine somatotropin (rbST) in milk [24]. Briefly, UV light from LEDs embedded in a 3D-printed smartphone attachment was used for excitation of quantum dot fluorescent labels used to visualize the amount of rbST. The images, collected with the rear camera, were then corrected by a developed Android-based software on the SBD and used to estimate rbST concentration. The system was compared with a flow cytometry reference method and obtained excellent agreement. The usefulness of such devices has equally been demonstrated for the detection of infectious diseases. Laksanasopin et al., has developed and compared a SBD for the simultaneous detection of syphilis and HIV within 15 min [25]. In this system microfluidic channels are coated with antigen recognized by marker antibodies present in whole blood samples of HIV and/or Syphilis patients. Whole blood samples are flown through the channels (using a hand driven vacuum pump), followed by Gold labeled IgM antibodies held in a chamber in the microfluidic cassette. Then, silver reagent is added to amplify the signal and the optical density is measured. This device was compared to laboratory-based tests in a small (*n* = 96) clinical trial in Rwanda and obtained excellent results. Finally, Priye et al., has recently developed a multiplex SBD for the detection of zika, chikungunya and dengue viruses. The authors use an isothermal PCR technique (reverse-transcription loop-mediated isothermal amplification) for fluorescent detection of the RNA viruses. The system is integrated in a small 3D printed box fitted with an excitation source and emission filters and powered with a 5V USB power source. Spectra are taken with the smartphone rear camera and analyzed using a developed smartphone app equipped with an algorithm to analyze the fluorescent signal. Again, it was shown that the SBD was capable to detect the targets in crude matrixes (blood, urine and saliva) with similar performance to bench-top devices [26]. Such techniques, embedded within a simple compact device that does not require extensive training before use, could potentially be used to scan for signs of stress, detect opportunistic microbes, keep track of an astronaut’s metabolism and perform preliminary in situ scans for extra-terrestrial life. Moreover, hyphenated biosensors, combined with immunosorbent assays, have been proven to work both under microgravity and Martian gravity [27].

### 1.3. Obstacles to Overcome to Enable SBD Use in Space

However, most SBDs have been developed for implementation on earth. In order to optimize a smartphone based device (SBD) for use in space travel special considerations are required with regards to the construction of the device such as the principle of detection, the biorecognition element and the sample type to be applied. One particular concern can be the degradation of protein-based recognition elements (antibodies/enzymes) used for bio-recognition. Thus, long-term stability to ensure functionality of the sensor throughout the trip is vital. Another possible obstacle for piggybacking SBDs for deep space analysis is the lack of protection of the electronics of the SBD from galactic cosmic rays (GCR) and solar particle events (SPE). Luckily, the protective shielding integrated in a crewed spacecraft might mitigate the need for additional electronic shielding. Indeed several commercial off the shelve (COTS) devices are being tested and used on the ISS with interesting results [28], including a COTS device using smartphone software (mobiPV) [29]. However, GCR and SPE outside of the earth’s magnetic field might still cause damage to such devices in deep space. Thus more research in the use of novel shielding materials, like doping polymeric casing with carbon nanotubes [30,31], or nanometals [32] would be useful to ensure the safe use of COTS devices in those settings. Another issue might be impaired functioning of REs such as proteins and DNA due to radiation damage. However, recent work has shown that proteins [33], including antibodies in protein arrays [34,35], as well as DNA based aptamers [36] show little to no loss of function at radiation levels which are orders of magnitude higher than the levels measured by the Curiosity rover on its mission to Mars [37]. Nonetheless antibody storage conditions remain stringent and long term storage can lead to reduced activity of antibodies [33]. Moreover, a study on radiation resistance of gDNA and primers showed PCR function inactivation at 180 Gy exposure to proton radiation at SPE energy levels [38]. This suggests higher radiation sensitivity for nucleic acid based systems as observed in [36]. Thus the use of more stable, synthetic REs might further increase the shelf-life of such devices and enable long term storage in less stringent conditions as needed for SBDs with protein based components. The aim of this review is to provide a guide to the presently developed SBDs that could prove utile, after limited adaptations, for their implementation in long-term space missions. Firstly, the possibilities to monitor human health with these devices will be discussed with an emphasis on the screening for infectious diseases, viruses, and the monitoring of biomarkers indicating the state of a person’s health, e.g., stress and immune response levels, as well as options to monitor the external environment for the presence of pathogens. In each of these sections effort is made to identify which are the most pressing risks that could be addressed using, or adapting, an existing SBD and which requirements such a device should meet to be utile for deep space missions by using information gathered from the human research roadmap (https://humanresearchroadmap.nasa.gov/Risks/). Secondly, the possible weak points of these biosensors for in situ analysis in space will be critiqued including the choice of the integrated recognition element (RE) and the use of synthetic biology to obtain shelf-stable reagents. Finally, a short synopsis will focus on the predicted further development of such devices for this purpose.

## 2. Existing SBDs Useful for Space Missions

### 2.1. General Overview of Available SBDs to Monitor the Crew’s Health

**Figure 1:**
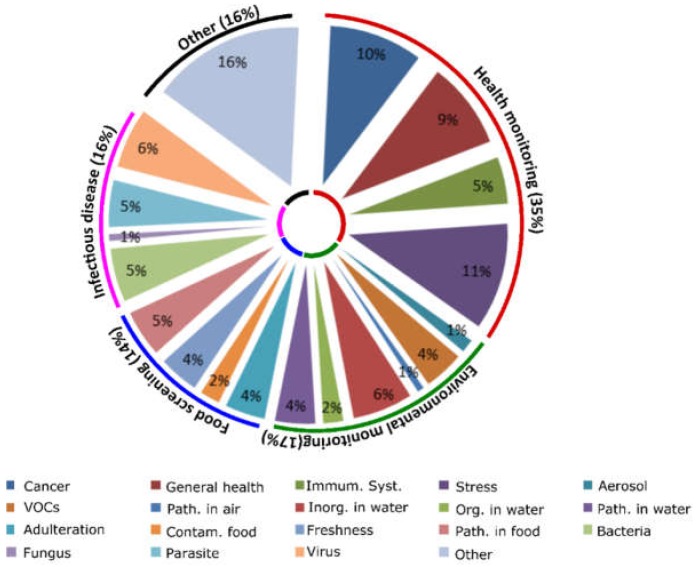
Classification of relevant SBD related literature. A pie chart showing the classification of the 148 articles kept for thorough analyses after the keyword searches. Groups (outer circle) are divided into subgroups (inner pie chart). Percentages indicate percentage of articles presenting the group or subgroup in relation to the total amount of articles kept for analyses.

The tasks that an SBD could perform during deep space missions are numerous and include monitoring the crew’s health, checking the health of any biological elements of life support systems, investigating microbiome health and soil health, and screening for signs of life upon landing. In this paper the main focus is to investigate the possibility to piggyback on existing SBDs for crew health monitoring during space missions. This specific application was chosen for two main reasons: (i) By measure of time priority. If SBDs are to be used for any application at the final destination they must first undertake the journey. Thus advantageously they might firstly be utilized to monitor crew health during that journey. (ii) Use of SBDs on a manned spacecraft instead of an unmanned craft, send ahead for life detection for example, implies protection of both the electronics and RE elements of the SBD from radiation. This limits the threat of malfunctioning and allows faster actual implementation of such instruments for use in space. This however, does not mean that the systems discussed here below cannot be useful for the other bespoken applications simply by changing the RE. Possible use of SBDs to monitor crew health was further classified into four groups: (i) Monitoring the general health of the crew (cancer, reduced immunity and signs of stress); (ii) screening food for freshness and contamination; (iii) monitoring the air and water quality on-board and (iv) diagnosis of infectious diseases. In order to classify the SBD reported in the scientific literature a keyword search in Scopus was conducted. The general query was: “(smartphone OR cell phone) AND (portable OR mobile) AND (instrument OR sensor OR device OR platform) AND (sensing OR testing OR analysis OR detection OR measurement OR monitoring OR diagnostics)”. The following terms were added to that query for the individual groups: AND (biomarker OR protein OR cancer OR stress) for group (i). AND (food OR foodstuff OR milk OR fruit OR cereal OR meal AND NOT allergen OR allergy) for group (ii). AND (air OR water OR environment OR volatile OR inorganic) for group (iii). AND (pathogen OR bacteria OR virus OR infection) for group (iv). This search yielded 155 original research articles for group (i), 78 for group (ii), 579 for group (iii) and 118 for group (iv). In group (iii) all articles related to computer science mainly discussing advances in app development and algorithm improvement were then excluded in order to focus on articles related to advances in mobile biochemical analysis which led to a reduction in that group to 199 documents. Overall, articles were considered in scope if the focus was on mobile biochemical analysis and if at least one of the possible group-specific application-related keywords was mentioned in the article as a detection target. After deletion of duplicates (5) a total of 550 articles remained for analysis. Based on the abstract content this number was condensed to 186 articles. Finally, after full article analyses and cross referencing, the following number of articles were deemed to enter into the scope of this review: 51 for group (i), 22 for group (ii), 27 for group (iii) and 25 for group (iv). Another 23 articles were deemed relevant but difficult to classify into one of these groups and were denoted “other”. Thus a total of 148 articles were reviewed and further classified into subgroups (Figure 1). There are approximately twice as many SBDs reported for general health monitoring compared to the other groups as perhaps more funding is available for research targeting all these issues including cancer (25% of the articles reported in the group) and cardiovascular and stress related problems (± 30% of the group). Moreover, the SBDs proposed to monitor cardiovascular and stress related disease often analyze the heart rate, using a variety of measurements such as pulse to pulse intervals and ECG, using a smartphone for the determination of stress levels [39,40,41,42,43,44,45,46], which is easier to accomplish than the specific detection of a pathogen. Some articles report the use of SBDs to monitor infectious diseases or to diagnose diseases that are unlikely to present during a space mission. However, these systems may still prove useful since they can be used to detect other targets by simply changing the bio-recognition element for detection of a specific contaminant. Interestingly, only one article was reported for the detection of fungi (Fusarium) using an SBD [47] indicating that more research in this direction is needed since fungi are major players in food spoilage [48] and several Fusarium species can produce toxic compounds [49]. The polyvalent group “Other” (16%) reports SBDs that use REs which require modification to become useful such as a printed paper assay for the quantification of streptavidin [50], or articles difficult to classify into a group as applied to diverse applications.

### 2.2. SBDs for Health Monitoring in Space

General health monitoring can be performed by observing physical and biochemical parameters. Key examples are highlighted of SBDs for their current use in detecting stress, reduced immune response and general health monitoring and in cancer diagnosis of importance for prolonged space travel.

#### 2.2.1. Detecting Stress

The human research program integrated research plan identifies stress as a very real problem which can jeopardize the mission due to several factors (e.g., high workload, circadian desynchrony, elevated CO_2_ levels, radiation and diet and nutrition), and stresses the need for early detection mechanisms to monitor the mental and cognitive health of crew members [4]. Moreover, risk of elevated cardiac rhythm problems during space flights was identified as a red zone in the likelihood consequence (LxC) rating in the human research plan and it is planned to conduct more detailed in-flight heart rate measurements to better predict risks for environmentally induced cardiovascular disease and determine the causes of this (https://go.nasa.gov/2OEifZN). Evidently, stress, equally listed as a risk factor in the human research plan, can be a contributor to this risk factor. On the other hand the multimodal nature of stress makes early detection using one single parameter virtually unattainable [51]. In fact, information regarding cortisol levels, heart rate (mainly ECG and heart rate variability (HRV)) and behavior should be integrated [51,52]. Unfortunately, most SBDs use only the heart rate to monitor stress levels although there are some exceptions where additional data from motion activity, posture [53] and communication data [54] are being used. This development increases the prediction accuracy [54]. However, it does not absolve the need for proper psychological assessment. Extended psychoanalyses and treatment can however be tedious during a mission to Mars since it requires intensive dialog which is complicated due to the extended one way light time (approx. 14 min) between Earth and Mars. Thus, the use of mobile devices that use a multimodal approach for early stress diagnosis and at the same time offer self-help solutions can be a welcome complementary approach. The U.S. military has recently developed an SBD that uses such a complementary approach. The SBD uses multiple sensors to detect stress and other psychological health problems and is equipped with an option for self-help via an app [55]. Another interesting paper reports the development of an application designed to deliver breathing awareness meditation to reduce stress levels [56]. Although data are preliminary, such systems could potentially not only allow diagnosis but also help reduce stress on a prolonged space mission. Apart from these developments other devices for monitoring an array of health related parameters including ECG measurements, blood oxygen levels, body temperature, and sleep quality are already commercially available and their potential usefulness for a mission to Mars was discussed in a recent perspective [57]. Although the paper might overestimate the ease with which the futuristic suggestions could be implicated and does not critically compare the functioning of the mentioned devices, it does provide an extensive list of commercial devices that are potentially interesting to test. In addition, other commercial devices like Google Glass, albeit in a slightly adapted format, have been tested and found interesting to use during space missions as a mobile procedure viewer assisting astronauts during various operations while enabling full two way video communication [29,58]. Moreover, commercially available wearable devices like smart-watches are already used in space and potentially integrate most measurements mentioned here. The interest of NASA for such devices is showcased by the crowd-sourced astronaut app competition that was recently held by NASA and won by I. Calvo and J. Richard for the design of such a system (https://bit.ly/2N1MDNy). In addition, major interest in tricorder like personal health monitoring devices became apparent during the Tricorder X-prize competition (https://tricorder.xprize.org/).

#### 2.2.2. Detecting Reduced Immune Response and General Health Monitoring

The production of naïve T-cells, which form an important protection mechanism against opportunistic viral and fungal pathogens as well as latent viruses, has been shown to decrease in astronauts after space travel [59]. Moreover, this reduction in thymopoiesis was linked to increased amounts of glucocorticoids in plasma and urine. Interestingly, Geiger et al., recently suggested that cortisol levels increased by stress exposure can indeed negatively affect the immune response to pathogens [60]. Indeed, reduced immune response during space missions is a concern included in the human research program integrated research plan [4] and the need for in-flight evidence regarding this is identified as a gap in the program (https://go.nasa.gov/2Mctbkt). More research regarding stress and immune response interplay might reveal the cause of the observed reduced immune response during space travel. One interesting avenue to investigate the link between stress and immune response during space missions is monitoring the cortisol levels in saliva and to link this to CD4+ cell count. Recently, a competitive lateral flow immunoassay (LFIA) and horse radish peroxidase (HRP) conjugated cortisol was developed to detect and quantify chemo luminescence [52]. A 3D printed device shields the strip from background noise and operator variation thus creating a robust test that can be operated by the layman yet allows cortisol quantification directly in saliva with a LOQ of 0.3 ng/mL and a linear range between 0.3 and 60 ng/L. Cortisol levels in saliva vary depending the time of day but typically remain between 0.6 and 10 ng/L [52]. Thus the developed assay (which takes about 30 min) can reveal clinically relevant information. Interestingly, the performance of a variation of this device has very recently been tested aboard the ISS station (mission 52/53) using saliva samples from an Italian astronaut, further underlining the interest in such rapid tests for in situ monitoring of crew health, and the results are pending (https://go.nasa.gov/2Kb854L). Such a device, in combination with an SBD that can determine specific T-cell densities in blood, could then be used to further investigate this phenomenon directly in space while keeping tabs on stress levels and other major markers indicating reduced immunity or infection like reduced CD4+ levels. Indeed SBD based cell counting devices exist already including an SBD using fluorescent imaging cytometry [61] and another using magnetic bead ELISA [62]. The latter may be more fit for the purpose of rapid on-site cell counting since the device uses highly specific monoclonal antibodies and does not require extensive treatment of the blood sample prior to analysis, in contrast to the former. Moreover, since it has been shown in several studies using different detection mechanisms that SBDs can have a similar performance as bench-top reference methods [23,24,25,26], and since the discussed magnetic bead ELISA assay shows a CD4+ T-cell count accuracy of 97% at levels below normal (350 cells/µL instead of ~1000 cells/µL which is the normal level), it can be considered feasible to do such precise measurements with an SBD. Vitamin D (VD) is another important biomarker as levels have been reported to decrease during space missions whereby supplements are required to limit bone loss [63] and minimize effects on both the innate and adaptive immune response [64]. Recently an SBD has been developed that allows quantification of VD [65]. For this purpose VD was aminopropylated and immobilized on a glass substrate whereby antibody coated gold nanoparticles (Ab-GNPs) were allowed to bind to this substrate in a competitive assay (fewer Ab-GNPs will bind if free VD is present in the sample). Finally silver ion reduction on the gold surface of the Ab-GNPs bound to the immobilized VD allows for sensitive colorimetric detection by using a mobile application that applies the hue saturation brightness model to quantify VD at nanomolar concentrations. However, the method does hold some weak points such as a required lengthy 6-h incubation. Finally some interesting work has been reported on mobile devices to detect glaucoma i.e., a 3D printed retinal imager [66] and a pressure sensitive microchannel [67] to measure blood pressure behind the eye.

#### 2.2.3. Detecting Cancer

The detection of certain cancers can be performed by SBD through the cellular image analysis or the detection of biomarker indicators. An exact risk assessment for cancer due to GCR is difficult due to the lack of sufficient data [68]. However, compelling indications that ionizing radiation can increase the risk for melanoma [69] exist and that exposure to GCR and SPE during a Mars mission can increase the risk of skin cancer in astronauts by >1% [70]. Evidently, this does not mean that astronauts have a high risk of developing cancer during the mission but rather that the life-time cancer risk will be elevated due to the stay in deep space. This being said, the use of facile screening methods for melanoma and other cancers can still be considered desirable on long-term missions to ensure optimum crew health and allow early detection. Indeed developing technologies for risk mitigation and monitoring are mentioned as desirable in the human research roadmap (https://go.nasa.gov/2KSjIt8). Several interesting SBDs targeting skin cancer came to surface in the cancer subgroup detecting malignancies via image analysis [71,72,73,74]. Of these the most complete system uses deep convolutional neural networks (CNN) to diagnose keratinocyte carcinomas versus benign seborrheic keratosis and malignant melanomas versus benign nevi in a binary classification system [72]. The system was trained using a substantial dataset (129,450 clinical images representing over 2000 diseases), tested and found as proficient in its diagnostic capabilities as skilled dermatologists. However, such a system does require the mobile device to be fitted with CNN which remains difficult since CNN requires considerable computing power. However, this situation might change in the future since graphical processing units (GPUs) are becoming more common in mobile devices. These GPUs have parallel processing capabilities which can be exploited to accelerate CNN computations on mobile devices. Moreover, an open source, GPU accelerated, library has recently become available on github [75]. Apart from this there is also a neural compute stick (Movidius) available on the market which shows promising results for the use of some CNN on low power devices [76]. This being said, mobile GPUs remain constrained for the use of very deep CNN networks and much work remains to be done on the development of mobile devices for the use of such sophisticated machine learning techniques [77]. An alternative to computed image analysis is visual microscopic analysis via a SBD microscope [78]. Here sensitivity and specificity of a SBD microscope was compared to a conventional light microscope for the expert analyses of dermatopathologic samples. It was found that the SBD had a similar performance as the conventional microscope except for the diagnosis of malignant melanoma where the sensitivity was only 60% but with good specificity (99.9%). However effective, this method still requires expert knowledge for diagnosis and thus requires the images to be sent back to earth. In theory this is not a problem since one-way light time (around 14 min) is not limiting for sending such data between earth and Mars although this delay can quickly add up to hours if active guidance of an expert is required while acquiring images. Thus, it may be more interesting to limit sending microscopic images that have popped up interesting during a preliminary screening test to limit unnecessary data analysis by experts. To this end it might be a more fruitful approach to use the microscope to gather images that could be further processed using image analysis. In this manner more direct analysis with less background noise and variables could also reduce the need for algorithms that require excessive computing power. Another way to reduce background noise might be to use a SBD spectrometer [79]. Such a system could potentially prove useful if enough data is collected to build a solid database for chemometric analysis. Indeed NIR spectroscopy with commercial smartphones is already coming available making such an endeavor more feasible (https://bit.ly/2Kong71). An SBD to detect ovarian cancer using a microchip ELISA to detect human epididymis protein (HE4) in urine [80,81], as well as prostate cancer using a microchip ELISA targeting prostate specific antigen (PSA) [82]. The latter SBD uses magnetic nanoparticles and a magnet rather than pumps thus simplifying the design. Moreover, the surface to volume ratio is thus increased which has reportedly reduced the analysis time to 30 min in contrast to 5 h for the assay. In addition, a SBD using a spectrometer to detect Interleukin 6 (IL-6), a biomarker for several cancers, using conventional ELISA [83], and a SBD using microfluidic dielectrophoresis combined with image analyses on a smartphone camera to count MCF-7 breast cancer cells in culture media [84] have been reported. Apart from these systems other intriguing SBDs exist that could be used for monitoring the crew health. The target, detection method and pros and cons of these systems are illustrated in Table 1.

### 2.3. Environmental Monitoring

Environmental monitoring including both water and air quality both whilst on board the spacecraft and on arrival in Mars will be an important element of consideration for a SBD to be valuable.

#### 2.3.1. Inorganic and Organic Compounds in Water

The storage of drinking water on a prolonged space mission must be economized to avoid adding unnecessary weight to the spacecraft. However, recycling water from urine, air humidity and hygiene water can lead to higher amounts of toxic metals due to leaching from metal coatings and filter resin failure and cause hazardous levels of multiple toxic metals in the water supply calling for sensitive detection of these metals at the ppb level [95]. SBD sensors have been reported for the detection of lead(II) ions [96,97] with LODs around 20 µg/L which is 2 fold below the requirements for regenerated potable water aboard the ISS (http://emits.sso.esa.int/emits-doc/RD5-ITT-1-5247.pdf). Unfortunately one of the methods is based on gravitational force [96]. Three SBDs are reported for Hg^2+^ quantification [98,99,100] and one for fluoride quantification [101]. Of these, one is especially [98] interesting since the LOD reported is almost 10 fold lower than bespoken requirements for potable water on the ISS (0.28 µg/L in [98] versus 2 µg/L aboard the ISS). Moreover, the testing time is limited to 20 min, and the sensor size is under 5 cm thus responding to the goal of reducing human systems resource requirements stipulated in the Human Research Plan-47052 revision E. However, none of these systems were used for the multiplex detection of toxic metals. Wang et al., used a paper fluidics device that uses stacked paper layers fitted with channels bordered by hydrophobic walls and adhesive tape to construct a device allowing the detection of 4 metals in 16 zones [102]. The system was tested for Cd, Ni, Cu and Cr using selective chromogenic reagents for a colorimetric readout that was then quantified using a smartphone camera and application. The authors found that quantification in the low ppm level was possible using this setup. Although sensing at ppb was not achieved the sensitivity could be increased by using other techniques for the detection like the immune detection of metals using HRP, GNP or quantum dots (QD) for enhancement while keeping the design of the device. As for the detection of organic compounds in water three SBDs were described including one that uses electrochemical detection of nitrate in water at the ppm level [103], one using colorimetric detection of catechols in river water [104] and finally one using an acetylcholinesterase inhibition assay to detect organophosphate pesticides in natural water resources [105]. Of these, one is especially [103] of interest since its LOD is 5 fold lower than ISS requirements (0.2 µg/L versus 10 µg/L respectively), while keeping the sensor mass at ~65 g and analysis time around 1 min.

#### 2.3.2. Aerosols, Pathogens and Volatile Organic Compounds (VOCs) in Air

**Figure 2:**
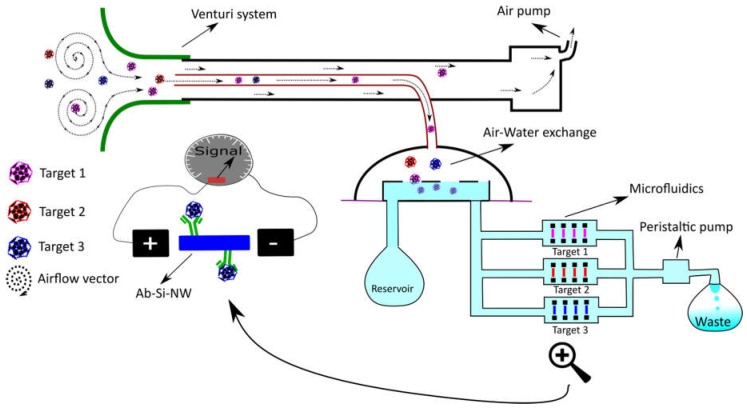
Schematic representation of a multiplex system for pathogen detection in air. The system depicted here follows the same principle as suggested in [100]. To this principle we added a presentation of enhanced aerodynamic reach as developed by [102] in combination with a simple microfluidic system to reach multiplex detection of several targets (as presented by pink, red and blue color particles).

Three existing SBDs have been described for the detection of small particles in air [106,107]. One focuses on the detection of particles on a miniaturized aerosol filter via subsequent image analysis of the observed color change and is effective to measure particle (mainly black carbon) concentrations but not particle size [106], an important parameter to determine particle carcinogenicity [108]. Another SBD, developed by the Ozcan group and termed c-Air [107], uses computational lens free imaging and machine learning to calculate particle size and distribution. In brief, the camera registers the holograms produced by the captured particles on a sticky surface. An iterative particle-peeling algorithm (which takes into account the generated twin image artifact and corrects for it) is then used to reconstruct the particle size from the interference patterns. Finally machine learning is used to further avoid the measurement of false positives. The system, which has a cut-off at 1.4 µm, was tested and found proficient when compared with a conventional device (BAM-1020, Met One Instruments, Inc., Grants Pass, OR, USA). Moreover, c-Air works at a ±15 times higher debit (amount of air analyzed per time unit) as other portable devices [107]. Moreover, the system would fit requirements (particle size ≤ 10 µM) described in NASA-STD-3001, VOLUME 2, REVISION A and adheres to the requirements for such in flight analysis devices (reduced mass, analysis time and power use) mentioned in the Human Research Plan-47052 revision E. As for the measurement of VOCs several SBDs have been developed. Chen et al., [109] developed a system that uses a porous graphitized carbo-pack fitted with a tungsten heating wire that enables pre-concentration of VOCs followed by sudden release at 300 °C. A small GC-column (varying from 4 m to 19 m depending on sample complexity) is used for separation. Detection is achieved via quartz mechanical resonators fitted with molecular imprinted polymers (MIPs) leading to the selective detection of a number of mono-aromatic and alkyl hydrocarbons at the ppb level. The entire procedure (from pre-concentration to detection and flushing the system) only takes a few minutes and has proven efficient in real-life situations making this an attractive portable method to detect VOCs. Finally, one SBD has been reported that enables the detection of pathogens in air (influenza; H3N2) [110]. The detection of this pathogen is especially interesting since the majority of infectious disease incidents reported among approximately 742 crew members in 106 space missions were fever/chills and flu-like illnesses (11 out of 29) [111]. The system uses antibody functionalized silicon nanowires (Ab-Si-NW) in microfluidic channels to detect conductance changes created by the binding event. Information regarding air quality is then displayed on a smartphone through wireless connectivity [110]. Such a system however only works in a conductive medium such as water and not in air. Thus the authors used an electrostatic air sampling system that allowed transferring aerosols into hydrosols which could then be transported to the Ab-Si-NW via microfluidic channels. This innovative system is a prime example of opening up the real-time sensitive water-world of Ab-based label free sensing to the detection of pathogens in air whereby it may be made multiplex by splitting up the microfluidic channel before detection. Such capability would potentially be very facilitating for space missions and could be remotely monitored on a computer. Moreover, the aerodynamic reach of such a system could be increased significantly by using a Venturi system for aerosol sampling [112]. In such a set-up the inlet tube used for air sampling could be reduced in diameter (from 16 mm in the original setup to about 5 mm) thus allowing the reduction of the water volume used for hydrosol formation while improving vapor collection which could lead to a higher concentration of hydrosols in the microfluidic channels, improve the detection limit and limit water use. Figure 2 shows a schematic of this futuristic device. Evidently, the device depicted can transfer information to a SBD for data processing while stationary, a system which was used by the authors for data processing, or could even be used as a portable device due to the reduced need of water for hydrosol formation.

### 2.4. Food Screening

Testing for microbial food contamination currently requires sample return and identification on Earth using culture-based methodology. Utilizing such a system in-flight on long missions is unfit for purpose due to the limited shelf life and mass of the consumables and other methods should be considered (https://go.nasa.gov/2P5XdUK). A recent review has already discussed the subject of SBDs for the screening of food quite thoroughly [19]. The most relevant articles on the testing of food freshness and screening for pathogens in food by SBDs are highlighted. This selection was chosen, excluding allergens and chemicals since the detection of these could be done prior to the mission whilst problems due to contamination with pathogens can emerge during the mission. Regarding food freshness most SBDs are used to determine the quality of fresh fruits using portable spectroscopy [113,114,115]. However, one interesting article focuses on the discrimination and semi-quantification of volatile amines emitted by microorganisms indicating rot [116]. In this article cellulose acetate membranes were spotted with 5 pH indicators. The membrane was then exposed to the amines that represent typical metabolic products from protein degradation by microorganisms. Red green blue (RGB) values were extracted and used to generate scores for a principal component analysis (PCA). The first 2 components of the PCA managed to explain over 72% of the variance (*n* = 4) indicating good separation of these VOCs. In a separate experiment the authors also managed to explain 85% of the variance between several biogenic amines (tyramine, putrescine, cadaverine) in a proof of principle test using analytical standards. Thus this simple test might prove useful to check the quality of rehydrated, lyophilized food after long term storage. Apart from this work there have been several reports on the detection of *E. coli* [117,118,119,120] and Salmonella [121] individually and *E. coli* and Salmonella together [122] in various fresh food products. Of these, three articles reported a detection limit at 10 CFUs or lower in real sample matrix (milk, yoghurt or egg) [117,119,121]. Thus these sensors show promise to be used on long space missions since they approach the limit of 0 CFU per food sample set in NASA-STD-3001, VOLUME 2, REVISION A.

### 2.5. Infectious Disease Detection

As for infectious diseases, many reports focused on the detection of infectious diseases unlikely to occur during any space mission (e.g., malaria [123,124,125], HIV [25,126,127] schistosomiasis [128,129,130], tuberculosis [131,132], and leprosies [133]). Nonetheless, these systems could be adapted for the detection of other infectious diseases. However, some reports of SBDs focused on the detection of HV [134,135,136]. Of these systems one uses fluorescent imaging as a detection method and thus there is a requirement to first label the virus particles for detection [136]. A second system is based on the detection of virus DNA by measuring the changes in optical density in a DNA-GNP solution specific for HV DNA upon HV addition. The system is promising but remains at a proof of principle stage for the moment [135]. The final system however, developed by the Ozcan group, has been thoroughly tested utilizing real clinical samples and proven to attain over 98% accuracy [134]. This system uses standard ELISA tests, in a 96 well plate. The wells are illuminated by LEDs and light is transported from each well to the smartphone camera via optical fibers. The data is then remotely interpreted using a machine-learning algorithm. Although this system is portable and well beyond the proof of principle stage, it would need further simplification to make it suitable for non-expert use upon a spacecraft. Overall, the majority of SBDs currently developed for the detection of infectious diseases focus on diseases unlikely to develop during space missions (malaria, schistosomiasis, HIV, etc.). For space missions however, the focus should be on microbial infections known to occur during space missions such as HV, urine tract infection and subcutaneous skin infections https://ntrs.nasa.gov/search.jsp?R=20140002769. In fact, a list of recommended specific infectious disease targets to screen for during deep space missions can be found in [111] and include meningococcus, pneumococcus, typhoid and several fungi.

### 2.6. Other, Unclassified SBDs

Many interesting technical papers describing the development of novel smartphone sensors fall within this group. Two describe the development of mobile spectrometers [137,138]. Another describes the development of an SBD with image resolution beyond the pixel size using lens free microscopy [138]. Fluorescent microscopy is evaluated also with the invention of a QD based Förster resonant energy transfer (FRET) SBD [139]. Another technique [140] describes the integration of an optical sensor into the touchscreen of smartphones by fabricating an optical waveguide just below the screen surface. The system is interesting because it allows the measurement of changes in the refractive index of liquids directly from the screen surface opening up the door to direct surface plasmon resonance (SPR) without the use of additional add-on devices. Although these SBDs can be promising in the future, none of them have been tested on real-life examples. Other SBDs, some of which show remarkable detection sensitivity, have been tested on targets from several groups. The targets and detection methods of these SBDs are illustrated in Table 2 together with their strengths and limitations.

## 3. Limitations of the Smartphone for In Situ Analysis in Space

### 3.1. Novel Recognition Elements

The RE used in a biosensor has a great influence on the price as well as the shelf life, selectivity and possibility of reuse of that sensor. The latter is especially of importance if quantitative, more expensive, SBDs are required for use during a space mission. Thus an informed choice regarding the RE to use for a certain type of SBD is paramount. The development, possible improvements and lurking pitfalls for effective sensor development using a variety of REs including antibodies [147,148], aptamers [149,150], MIPs [151,152], enzymes (divided in detection via substrate conversion [153,154] and inhibition of this conversion [155], riboswitches [156], affibodies [157] and cell-based biosensors (CBBs) [158] were recently reviewed. Table 3 lists each of these REs together with a summary of the findings of these reviews regarding the pros and cons of each RE. However, upcoming REs that can potentially entail major advancements in the development of rugged portable sensors with a longer shelf life and with expectations to be more capable to withstand harsh conditions, have not as yet been critically reviewed elsewhere and are described herein.

### 3.2. MIP-Aptamer Hybrids

**Figure 3:**
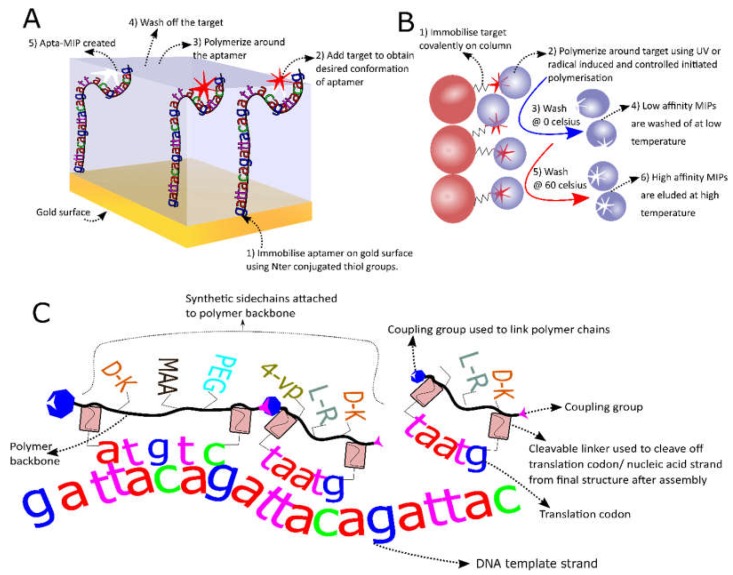
Novel recognition elements (REs). (**A**) This Schematic shows Apta-MIP hybrid fabrication steps where the aptamer is first immobilized on a gold surface after which the target is added. Next electropolymerization is followed by a washing step to produce the final hybrid; (**B**) Solid phase MIP production. The principle of solid phase MIP production starts with the immobilization of the target and ends with stringent washing to eluate the high affinity MIPs after whereby step 2 can be repeated several times; (**C**) A schematic showing the principle of enzyme free translation of polymers. The system that can be used to achieve the enzyme free translation of polymers carrying a pool of various side chains (here methacrylic acid (MAA) polyethylenglycol (PEG), 4-vinylpyridine (4-VP) and some amino acids with varying chirality are shown but others can be used).

Aptamers have been used for the construction of biosensors since SELEX was invented in the 1990s [160]. Although, the development of Aptasensors is promising, there are a few drawbacks to the system such as degradation of the aptamer by nucleases and lower affinity compared to antibodies [150]. One interesting sensor that was recently built to overcome these limits is a novel aptasensor that uses an aptamer covered with a layer of MIPs (Apta-MIPs). This RE has been developed for the electrochemical (EC) detection of PSA [161]. Briefly, a gold surface was functionalized with a PSA specific aptamer after which PSA was added to allow the aptamer-target complex to be formed. Then electro polymerization of dopamine was initiated around the complex. Finally PSA was washed away leaving the aptamer in a stable polymer layer which protects against degradation. The group showed that the LOD (1 pg/mL) was three times better than the LOD of the aptamer alone. Shortly after a similar technique was used by another group for the detection of enrofloxacin, (a fluoroquinolone antibiotic) via up-conversion fluorescence [162]. Again a very low LOD (0.04 ng/mL) was achieved as well as a good quantitation limit (0.12 ng/mL) with a relative standard deviation of 1–5%. These sensors are sensitive, stable and need less template as MIP sensors (a common obstacle for MIP production) [151], thus combining the best of 2 worlds. Finally another group showed that the polymerization of the fragments of an adenosine specific aptamer can rescue the binding of these fragments for adenosine, which was virtually absent for the free individual fragments [163]. This work opens the doors to new MIP fabrication using nucleic acids as functional monomers; the development of which is much needed to further boost MIP development for more diverse targets [151]. The basic production mechanism for Apta-MIPs is shown in Figure 3A.

### 3.3. Solid Phase MIPs

The use of MIPs is summarized in Table 3 and more information can be found in reviews [151,152]. However, one particular type of MIP, which is believed to be especially interesting for SBD development (Solid phase MIPs or MIP nanoparticles (MIP-NPs)), was not treated in any identified review. Thus, these will be reviewed here in more detail. NP-MIPs are produced by covalently fixing the template molecule to a substrate (glass beads) after which initiated polymerization occurs around this fixed template. These “plastic antibodies” were first developed by Poma et al., for melamine, vancomycin and a model octapeptide [164]. MIP-NPs are promising because they show high affinity (K_d_ in the nM range) at a lower production cost, improved shelf life and stability compared to antibodies [165]. MIP-NPs can be further functionalized with fluorescent groups, thiol groups, electro active groups or molecules with antifouling properties for further biosensor development [166]. Figure 3B shows the basic steps for MIP-NP construction. The system was also used for the construction of MIP-NPs targeting histamine [165,167], vacomycin [168,169] and fumonisin B2 as a first MIP-NP targeting a toxin [165]. Furthermore, the performance of the latter was compared with monoclonal antibody (mAb) performance in an ELISA assay and showed a 3X lower LOD (6.1 pM for the MIP-NPs in comparison with the mAb at 25 pM) as well as an improved linearity range [170]. Moreover, the system can be automated both for MIP synthesis in solvents [164] and in water [171]. The latter is preferable if proteins or other biomolecules are used as a template since buffers can be used that conserve the natural steric conformation of the templates [170,171]. Production in organic solvents is better for small molecules where H-bridge formation between template and functional monomer is important [151]. A protocol for the development of these MIP-NPs in both media [172] as well as a protocol describing how to perform enthalpy calculations of the monomer-template complex [173] were recently published. Unfortunately, the latter is based on software (Sybyl) that is no longer available. Although these developments are very interesting it must be stated that MIP-NPs generally require more template than aptamers or antibody production. Although this problem might be overcome by the use of rational design and multiple reuse of the covalently linked template in solid phase production, laborious and costly optimization might be needed to attain this goal. Moreover, non-covalent free radical or UV initiated polymerization used for MIP-NPs can attack double bonds in the template molecule, thus covalently linking template and MIP-NP. This can potentially make it very hard to remove the template molecules containing alkene groups.

### 3.4. In Vitro Selection of More Diverse Polymers

In order to block nucleases and increase structural diversity, thus increasing the chance of better target recognition, aptamers are often modified with non-natural nucleic acids or peptide chains [150,174]. Recently some intriguing advances in this area have been made like the use of peptide nucleic acid (PNA), a nucleic acid sequence with a peptide backbone that cannot be degraded by nucleases nor proteases with lower salt sensitivity and greater affinity to its target nucleic acids as its DNA counter sequence [175], directly for the in vitro selection process using PNA transcription enzyme [176]. A similar technique was used by the Chaput team, which used enzymatic transcription of threose nucleic acid (TNA) for the in vitro selection of a TNA aptamer targeting thrombin [177]. The limitation of these techniques however is the enzymatic transcription step which ultimately limits the amount of possible polymers that can be used for in vitro selection. Recently, a novel system that uses enzyme free translation of non-nucleic acid polymers (NNAP) was developed [178]. This system uses a quintuplet codon, much like a T-RNA, to proximate monomers to each other using a random ss-DNA pool for template (Figure 3C). After in vitro selection the sequence of the polymer can be recuperated via a fluorescent PAGE assay and ESI-MS [178]. Although this system has not been used directly to develop an SBD yet it does hold great potential to develop more diverse polymers targeting a structurally highly diverse group of compounds more efficiently.

### 3.5. Cell Free Synthetic Biology, the Answer to Long-Term Storage?

Some of the issues for deep space missions and extra-terrestrial settlement are the cost and impracticality of shuttling goods to the destination. Indeed it was estimated that the transport of 1 unit of goods requires 99 additional units of mass in fuel to get the product into space [179]. Moreover, the cost is approximately $10,000 per pound payload [180]. Thus, solutions have been sought to limit payload including self-sustaining life support systems [181] and bio-printing for onsite food production [180]. To this end use of synthetic biology has often been suggested as a viable solution [179,180,182]. An interesting use of synthetic biology is cell free protein production. Here, reagents e.g., DNA, transcription and translation machinery, are mixed to produce metabolic products and proteins in a cell free reaction chamber. The main advantages of such a system are the high production rate, yield, product pureness and ease of gene editing. Post translational modified (PTM) protein production however, such as disulphide bridge formation and protein folding (paramount for antibody production), remains difficult [183]. Luckily, different strategies to surmount this problem exist, such as changing the redox potential using glutamate [184,185], or adding chaperone proteins to the reaction mix [186]. A detailed protocol for cell free protein production of reporter proteins is freely available [187]. Another big advantage of such a system, which is especially valid for deep space missions, is the possibility to transport the reagents in lyophilized form at room temperature (RT). This technique was recently used to manufacture a low cost (less than 1 $ per test), user friendly, paper based colorimetric test for the detection of Zika virus [188] as well as Ebola virus [189] from *Escherichia coli* based extracts. It was shown that these systems can remain stable over 1 year at RT which greatly improves storage facility. Other work from the same group also demonstrated the production of antimicrobial peptides, cancer biomarkers (HER2, CEA5), fluorescent protein (mCherry), cytokines, small molecules, *Clostridium difficile* exotoxin (TcdA) and several antibodies using freeze-dried cell free *E. coli* based extracts [185]. However, these systems remain quite novel and not all reports show similar performance. A report from Smith et al., for instance, shows that protein activity can indeed be partially preserved at sub-optimal temperatures (4 °C) although it does decrease drastically after only 90 days of storage at RT even when sucrose is added as a lyoprotectant [190]. Nonetheless the potential advantages that these techniques have for long space missions justify further development in this intriguing field.

## 4. Conclusions and Outlook

**Figure 4:**
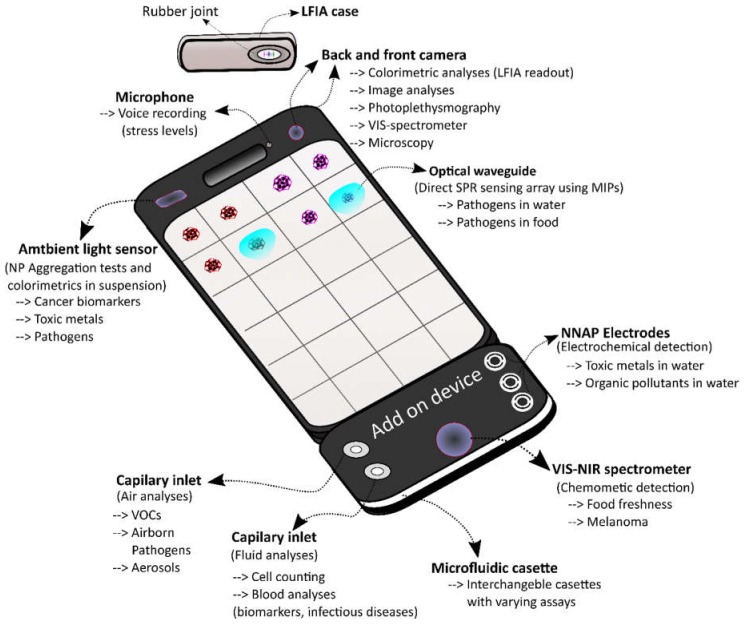
The galactic SBD. This figure shows a futuristic SBD that incorporates novel sensing technology with synthetic REs directly into the smartphone as well as using an add-on device equipped with interchangeable microfluidic cassettes. Possible uses and basic functioning mechanisms of these sensors are indicated.

A plethora of SBDs for human health related purposes have been developed in recent years. These compact and novel sensors stretch from simple sensors that allow the detection of stress through heart rate measurements using photoplethysmography to sophisticated biosensors using microfluidics and isothermal nucleic acid amplification methods with single copy DNA detection limits. In order to view and improve those currently available, the cornucopia of sensors were divided into four groups based on their usefulness for the monitoring of the crew’s health and further divided into subgroups as the most promising sensors for use aboard a spacecraft on a mission to Mars. Using this classification system and building on previous work mapping out LOC use in environmental monitoring [18] and food analyses [19,20], it became apparent that several SBDs for the monitoring of stress levels, skin cancer diagnostics, water screening for toxic metals, and infectious disease monitoring have been developed well beyond the proof of principle concept. However, different targets, more fit to the needs for microbial detection in space, would be necessary for the latter as pointed out in [111]. Moreover, the development of other groups such as testing for food spoilage (other than fruit) and scanning for airborne pathogens with SBDs is clearly lagging behind while demand for miniaturized devices for astronaut crew health monitoring is rising [191]. Thus more effort should be invested in these areas. Another observation is the prevalence of antibody based SBDs, in particular LFIAs with a smartphone readout that are often mentioned. Interestingly, LFIAs were previously identified as ideal systems for use in space [191] and thus LFIA SBDs might be the logical step forward. However, other more complex formats using unconventional REs have also been described herein as interesting alternatives. In particular MIP-NPs, MIP-Aptas and NNAPs have been identified as promising REs for space missions due to their increased stability, shelf life and protection against degradation. In summary, it is believed that several of the currently available SBDs could in theory be adapted for the use in space and aid in the monitoring of the crew’s health on a mission to Mars in a user-friendly fashion as light portable devices. When peering into the future, one could imagine an ideal SBD adapted for a prolonged space mission. This “galactic” SBD, would have to be equipped with a maximum number of sensors that are able to withstand harsh environments, without losing sensitivity, for a prolonged time as a primary requirement. Moreover, such sensors would ideally be integrated in the phone to maximize space, use shelf-stable components, have a user friendly “one step only” approach and work in (near) real-time to facilitate rapid and easy use by non-experts. A good sensor for the galactic, that potentially meets these requirements, is an integrated optical waveguide in the smartphone screen [140]. Such a system potentially allows direct multiplex SPR measurements on the screen. Covalently attached MIP-NPs could be used to enable sensitive sensing without degradation caused by proteases, temperature and pH variations and mechanical stress associated with touch screen use. This would in theory enable label free detection of multiple harmless analytes such as stress or cancer related biomarkers, by simply depositing a drop of sample on the indicated area of the screen. A limit for this technique would be the molecular weight of the target, which is correlated with the generated refractive index change upon binding. Moreover, it would be unfit to screen for pathogens. For the direct detection of smaller analytes an aptasensor, with electrochemical detection, could be used. Such a system is advantageous for small compounds since its sensing capabilities are based on the conformational change of the aptamer making the weight/electric resistance of the analyte less important. Moreover, a rapid one step approach can be achieved if a signal enhancer like methylene blue is attached to the aptamer [192]. Again, a stable synthetic RE like an NNAP would avoid degradation by proteases and nucleases and might enable direct analyses at the surface. An add-on device could be used for this purpose and could further be equipped with a VIS-NIR spectrometer/camera to allow spectrum analyses over a larger spectrum range and allow for the detection of more abundant compounds [115]. Moreover, the camera could be used for optical detection of a cornucopia of microfluidic assays such as fluorescent imaging cytometry or microfluidic dielectrophoresis for cell counting, magnetic bead ELISA for the detection of cancer or infectious disease markers or pathogens and an aerosol/hydrosol exchange system for the detection of pathogens in air. Microfluidic cassettes could be designed using a capillary plug-in so that multiple tests can be performed on the galactic by simply changing the cassette. This way the galactic could be made ready to perform even more tasks needed upon the arrival at Mars such as performing preliminary scans for extra-terrestrial life, or detecting the presence of essential nutrients or contaminants to realize Martian crop growth. Figure 4 shows an illustration of this futuristic sensing device indicating some possibilities for multiplex sensing. The envisaged compactness of such a system, combined with the vast arsenal of possible types of analyses that could be conducted by such a device, could greatly reduce the amount of space needed aboard. Moreover, analysis time, and thus workload (an ever persisting pressure on the crew), could be greatly reduced due to the multiplex nature of the “galactic” combined with the one-step only approaches mentioned. Keeping tabs on the concentration of multiple biomarkers in-flight might also improve our understanding of the effects of space travel on several biological phenomena and be beneficial for future missions. Thus such a system potentially provides interwoven benefits for the actual crew travelling to the faraway destination, and for those who will follow. Evidently, this ideal galactic sensing system remains fictional and may seem farfetched. However, technological advancement in the biotechnology sector is moving incredibly fast and the foundations for such a device have already been laid. This means that it might be more a question of perseverance and the will to dream and dare to turn this idea into a reality than anything else.

## Figures and Tables

**Table 1:** A list of SBDs developed to monitor general health features relevant for space missions.

Target and Device Working Conditions	Detection Method	Pros and Cons
Hemolysis in blood [85]. LOD: 1.39 mg/dL hemoglobin. Matrix: Plasma. System showed higher accuracy as conventional methods (Roche Cobas c501 and Siemens Dimension Vista 1500) and fast analyses time (10 min versus 4 h for conventional lab-based methods)	Colorimetric detection of free hemoglobin levels in plasma. Plasma is imaged and image-analyses is used to determine the amount of free hemoglobin levels present.	Pro: fast (10 min), cheap (few dollar), and relevant (astronaut anemia can be measured)
		Con: Blood separation based on gravitation in capillary
Cell density detection [86]. System able to distinguish between normal red blood cells (RBCs) and RBCs from anemia patient. It was suggested as method to detect low-density neutrophils as well but this was not tested.	Magnetic levitation. Cells in a capillary filled with a paramagnetic medium are placed between 2 rare earth magnets and their levitation position is determined solely by their density.	Pro: Fast and facile identification of astronaut anemia and other diseases that evoke cell density changes
		Con: Proof of principle only
Non-contact vital sign detection such as sleep apnea, pulse wave velocity measurements and respiration monitoring [87].	Doppler radar sensor. Integration of demodulation techniques and miniaturization (System on chip) to enable SBD detection.	Pro: basic vital signals can be remotely measured and analyzed
		Con: Experimental and not robust, sensitive for noise from movement
Tidal volume, V(T), estimator [88]. V(T) was estimated using a commercial spirometer for simple calibration. Method enables V(T) estimation with about 18% error compared to spirometer data.	Video analyses of chest movement	Pro: non-invasive monitoring of lung volume
		Con: Other simple and more direct methods exist as well
Mobile cell migration assay for neutrophil and cancer cell chemotaxis [89]. System achieves 3 µm resolution and was validated for detection of chronic obstructive pulmonary disease in clinical samples.	Test kit consists of a smartphone-imaging platform using microfluidic channels, LED illumination, emission filters and image analyses.	Pro: Neutrophil chemotaxis can be tested directly from a drop of blood.
		Con: System still at proof of principle stage
Detection of chronic obstructive pulmonary diseases [90]. System showed high correlation with breathing frequency and peak flow rate.	Resistance relative humidity sensor. Nanoparticle doped paper (NDP) resistance was measured during NDP exposure to breathe channeled through mouthpiece.	Pro: Quick way to detect chronic obstructive pulmonary diseases
		Con: System still at proof of principle stage
Quantitative clinical method for total protein, albumin, and hematocrit analysis [91]. Calibration curves showed good dynamic range and RSD values under 5%.	Colorimetric detection on polyester-toner, laser printed, microfluidic disks. Test enables both whole blood separation and component detection using SBD image analyses.	Pro: System is quick and fully integrated.
		Con: The system is complex (production costs)
Determine water-fat ratio in the body [92]. Method was compared to dual-energy X-ray absorptiometry (DXA) in healthy volunteers and showed a maximum absolute error of 6.5%.	Bioelectrical impedance analysis using a miniature multi-frequency impedance spectrometer for whole body impedance measurements.	Pro: Non-invasive, rapid and accurate
		Con: System still at proof of principle stage
Determine hemoglobin concentration and detect HIV virus [93]. System was validated in clinical trial (*n* = 38) showing 95% limit of agreement for hemaglobin and 95% sensitivity and specificity for HIV immune assay.	Microfluidic device with colorimetric detection to determine hemoglobin concentration and absorbance (silver enhanced precipitation of colloid gold) for HIV related antibody detection.	Pro: System is simple does not require expertise for use
		Con: System still at proof of principle stage
Urinary tract infection detection [94]. Application functions independently of room illumination and smartphone type (6 phones both Android and iPhone tested).	Colorimetric detection using image analyses. Device needs reference values for training set. Device is equipped with auto-localization to classify and detect ± 100 spots of 12 biomarkers simultaneously	Pro: multiplex detection of 12 biomarkers within one picture
		Con: semi-quantitative only, varying illumination can effect results

**Table 2:** A list of the SBDs that were not classified in any group since they have targets from various groups. Targets, detection methods, advantages and limitations of each SBD are highlighted.

Target	Method	Advantages	Limitation	Reference
*Escherichia coli*, Salmonella enterica, Rift valley fever virus with sensitivity close to single target copy. Method was validated using RT-qPCR.	Inhibition of DNA-paramagnetic silica bead aggregation, otherwise induced in longer strand DNA mixtures, by centrifugation after LAMP (a).	Single copy detection of DNA using simple device replacing fluorescent detection with simple aggregation assay measurable directly with SBD camera.	No guaranty regarding specificity in assay. Any short DNA amplicons will shield the beads from aggregation	[141]
Water born parasites, CD4+ T-Cells are detected in an 81 mm^2^ wide view with 10 µm resolution. An experimental protocol is included.	Fluorescent imaging flow cytometry using microfluidics, LED excitation and time-lapse video recording using the digital frames for cell counting. Also wide view microscopy using the smartphone camera is demonstrated.	Wide field of view for good diagnostics at low copy number and mobile cell counting.	Target must be fluorescently labeled prior to analyses	[142]
Multiplex (384) lateral flow protein micro array for clinically relevant biomarkers. Accuracy was 98% compared to established glass microarray for 26 antigen specific antibodies.	Paper based lateral flow protein microarray using biotin conjugated secondary Abs and anti-biotin coated GNPs	High multiplexing possibility and sensitive detection (30 ng/mL) in 10 min.	Multiple amplification steps can impede accurate quantification. High multiplexing can reduce signal to noise ratio.	[143]
DNA or RNA detection of multiple analytes in diverse matrixes (blood and water) using various microfluidic devices is described.	Microchip combining filtration, cell lysis, isothermal amplification and fluorescent detection for virus and bacteria.	Sensitivity and specificity comparable to conventional bench top methods	Complex matrix can impede enzyme assisted isothermal amplification	[126]
Human C-reactive protein (CRP) detection by sandwich ELISA, HRP detection for direct ELISA and BCA total protein estimation assays were performed for the SBD and compared to conventional microtiter plate readers (MTPR).	Standard ELISA tests read out by smartphone camera. SBD showed equal performance to conventional MTPR for LOD, LOQ, dynamic range, sensitivity and precision for all 3 assays.	Simple application using already existing established methods with low cost and miniaturized material.	Analyses requires same time frame and expertise as conventional ELISA	[144]
Carcinoembryonic antigen (CEA) (1) and (2), adenosine triphosphate (ATP). LOD for CEA was 6.1 pg/mL. LOD for ATP was 11 µM. Normal range of CEA is < 2.5 ng/mL and ATP roughly 1 mM. Thus mentioned LODs show usefulness’ of the device.	Inhibition of peroxide induced etching of nanoprisms and color change by presence of more Ab-NPs at high target concentrations (1). GNP aggregation inhibition by ssDNA stabilization after target binding with aptamer and dsDNA dissociation (2) (b).	Simple system using the ambient light sensor to detect the color changes in the suspension.	Complicated setup. Especially using dsDNA which dissociates to ssDNA (for GNP stabilization) and aptamer-target complex. The functioning of the system might be very dependent on the salt concentration in the matrix	[145]
Relative particle number densities determined in food (fat droplets in milk, yeast in water) and medical (RBCs in whole blood) matrixes.	ELS (c) with diode laser is used to create angular resolved scattering patterns which are imaged by the SBD camera. Mie theory is then used to calculate particle size.	Cheap determination of size distribution of particles in blood, yeast and milk.	Poor accuracy (±20 nm) and at proof of principle stage.	[146]

(**a**) Loop mediated isothermal amplification. (**b**) An inhibition assay where dsDNA (an ATP-aptamer and its complement) are incubated with ATP at 37 degree. ATP presence ensures structural change of the aptamer and avoids reformation of dsDNA thus preventing salt induced aggregation. (**c**) Elastic light scattering.

**Table 3:** A description of REs which illustrates the advantages and limitations of each.

RE	Description	Advantage	Limitations	Reference
Antibody	Specialized immune protein capable to recognize its antigen via a key-lock principle. Antibody antigen binding is based on Van der waals, hydrophobic and hydrogen bonds making it quite a stable complex.	Highly developed protocols exist, LOD often in pM range. Antibodies can often operate in quite varying conditions (pH, Salinity, complex matrix) and many protocols exist, making antibody based detection often the method of choice.	Production cost of monoclonal Ab is high. Protein can degrade limiting long-term storage. Setting up a reliable hybridoma line for monoclonal antibody is costly and can take years. Antibodies are primarily produced in animals.	[147,148]
Aptamer	Oligonucleotide designed to specifically bind its target (often upon conformation change) via subsequent systematic selection of the best binders available in a randomized pool of oligonucleotides. This selection process is called SELEX (systematic evolution by exponential enrichment). Many varieties of the process exist.	Developed protocols exist, LOD in the nm and even pM range is reported. Production is synthetic and cheaper as antibodies. Aptamer-target complexation often results in a significant conformational change of the aptamer which can be used as a label-free sensing principle.	Often binding specificity is sensitive to salt concentration. Degradation sensitive due to nucleases, hard to use in complex matrix.	[149,150]
MIP	Polymers with functional groups capable to interact with target functional groups are polymerized around the target. Next the target is eluded leaving a functionalized pocket behind to act with the target via a key-lock interaction principle	MIPs are cheap to produce if the target is not expensive. MIPs are very stable, leading to long shelf life. Detection limits in the pM range are reported but less common.	Washing out the template molecule can prove difficult. Target affinity can change between batches. Higher amounts of template is needed which can increase production costs.	[151,152]
Enzyme activity inhibition	The ability of an enzyme to catalyze its reaction is inhibited by the presence of a pollutant. The method is often used to detect organophosphorus pesticides. In such assays the enzymatic catalyzed conversion of a substrate to a colored product is often measured. Absence or reduction of the intensity of reaction indicates enzyme inhibition.	OPA (a), OPAA (b) and ACHE (c) enzyme inhibition assays are cheap and fast tests ideal for on-site screening. Especially OPH and OPAA enzymes are good candidates since they allow sensitive 1 step only detection. Moreover, genetically engineered recombinant enzymes of these groups exist and result in higher sensitivity.	Enzymatic activity can be reduced by many different compounds. Thus the specificity of this system can be compromised if real samples are used. Work remains to be done to further engineer OP and OPAA enzymes for optimal results.	[155]
Enzymatic substrate conversion	Enzymatic catalyzes of a compound leading to direct or indirect electron transport to an electrode used in electrochemical detection or conversion to a fluorochrome or colored compound for optical detection.	A wide variety of sensors based on this principle exist some of which like glucose sensors have proven to be fast, sensitive, low cost and reliable.	The inhibition of catalytic activity can lead to false negatives. Especially in matrices from patients containing ROS (d) and or inflamed tissues containing proteases capable to degrade the enzymes.	[153,154]
Riboswitches	RNA based system comprising 2 domains, a recognition domain (aptamer) and signaling domain. Upon recognition the conformational change frees an area of the signaling domain that can inhibit or promote translation of a protein or transcription of a reporter gene, triggering a fluorescent response. In some cases fluorescent response even occurs directly upon binding the analyte. These riboswitches are called fluorogenic riboswitches.	This system is very effective to enable small molecule induced gene regulation and can be used with synthetic aptamers to create fluorescent RNA based biosensors as internal validation for CBBs. Moreover, synthesis is synthetic and cheap compared to antibodies.	The best functioning riboswitches are prokaryotic. They will need to be adapted to use in eukaryotic cells to prevent rapid degradation of the RNA. For this non-natural nucleic acids, equally used for aptamer construction, might proof useful.	[156]
Affibodies	Synthetically constructed peptide scaffolds combined with a specific peptide sequence used as the RE. The Scaffold sequence (around 6.5 kDa) contains no cysteine and often stays the same. The variable region classically contains 13 amino acids and can be specifically engineered for a given target.	Smaller then antibodies thus closer to surface of transduction element leading to low LODs. Scaffold can be engineered to allow orientated immobilization. Absence of cysteine avoids artificial sulfur-bridge formation.	The method is relatively undeveloped. Some initial successes are booked but more research is needed.	[157,159]
CBBs	Living cells are integrated in the sensor. Their shape change, cell membrane damage or dead caused by interaction with the target are reported through optical or electrochemical detection.	CBBs have the unique ability to offer a measurable response to a pollutant related to actual physiologic responses of the subject to the substance.	The cells must be kept alive to function making long-term storage difficult. Many structurally different compounds can cause a similar response making downstream identification complex. Moreover, CBB sensors often require lengthy incubation and measuring steps in an incubator seriously limiting portability.	[158]

(**a**) OPH is organophosphorus hydrolase. (**b**) OPAA is organophosphorus acid anhydrolase. (**c**) ACHE is acetylcholinesterase. (**d**) ROS is reactive oxygen species.
